# Supplementary material for: Laboratory assessment of alternative stream velocity measurement methods
Source: PLoS One. 2019 Sep 6;14(9):e0222263. doi: 10.1371/journal.pone.0222263 (PMC6731056; doi:10.1371/journal.pone.0222263)
Supplement: S1 Table — (DOCX) [file pone.0222263.s001.docx]

| Calibration strategy | Training RMSE (cm/s) | Test RMSE (cm/s) |
| --- | --- | --- |
| Linear fit between uncalibrated and surface velocity; then multiply by modelled velocity ratio | 6.5 | 7.2 |
| Linear fit between uncalibrated and top 5 cm velocity; then multiply by modelled velocity ratio | 7.1 | 7.5 |
| Linear fit between uncalibrated and top 10 cm velocity; then multiply by modelled velocity ratio | 9.1 | 9.3 |
| Linear fit between uncalibrated and top 15 cm velocity; then multiply by modelled velocity ratio | 11.2 | 11.5 |
| Linear fit between uncalibrated and depth-averaged velocity; then multiply by modelled velocity ratio | 7.5 | 8.9 |
