# Supplementary figures and images for: Laboratory assessment of alternative stream velocity measurement methods
Source: PLoS One. 2019 Sep 6;14(9):e0222263. doi: 10.1371/journal.pone.0222263 (PMC6731056; doi:10.1371/journal.pone.0222263)

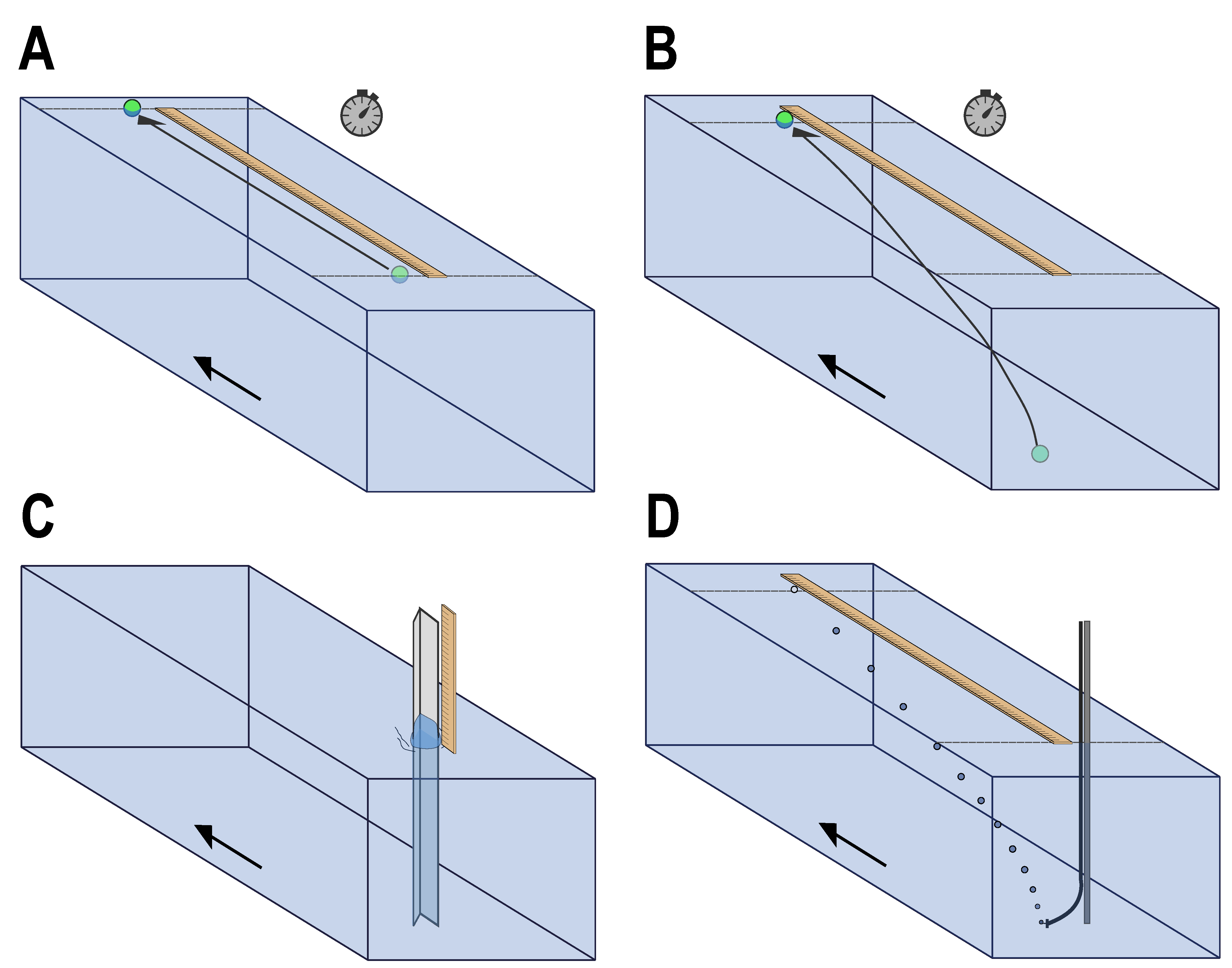

Supplement: S1 Fig — Subfigure A shows the surface float method, with the required materials of a water-fillable float, yardstick, and stopwatch. Subfigure B shows the rising body method, with the required materials of a water-fillable float, yardstick, and stopwatch. Subfigure C shows the velocity-head rod method, with the required materials of a velocity head rod and a ruler. Subfigure D shows the rising air bubble method, with the required materials of a bubbler and a yardstick. (TIFF) [file pone.0222263.s002.tiff]

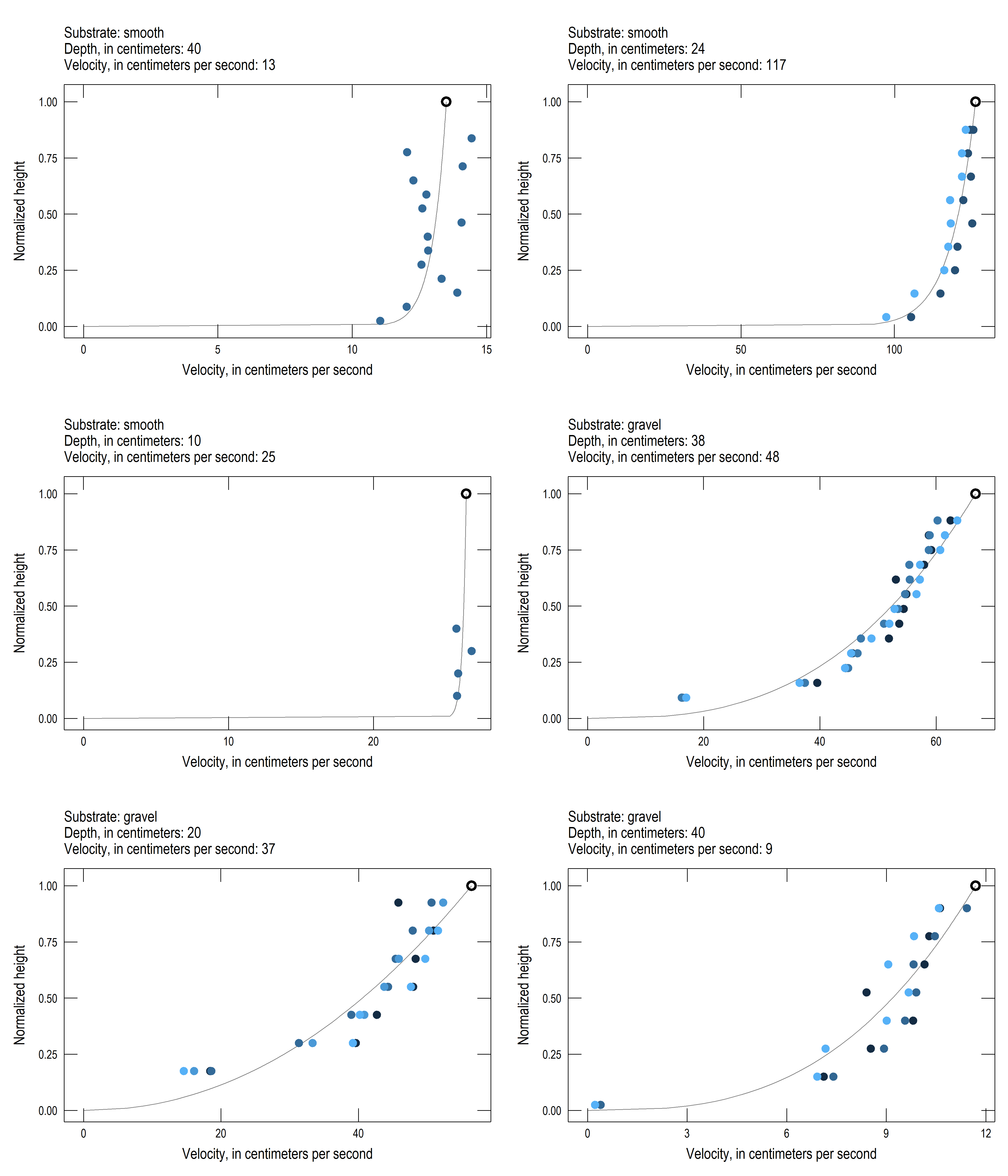

Supplement: S2 Fig — The blue dots show the velocity measured by the flume ADV. Different shades of blue are profiles measured at different times. A power law curve fit to the ADV measured velocities is shown as a black curve and the extrapolated surface velocity is shown as an unfilled black circle. (TIFF) [file pone.0222263.s003.tiff]

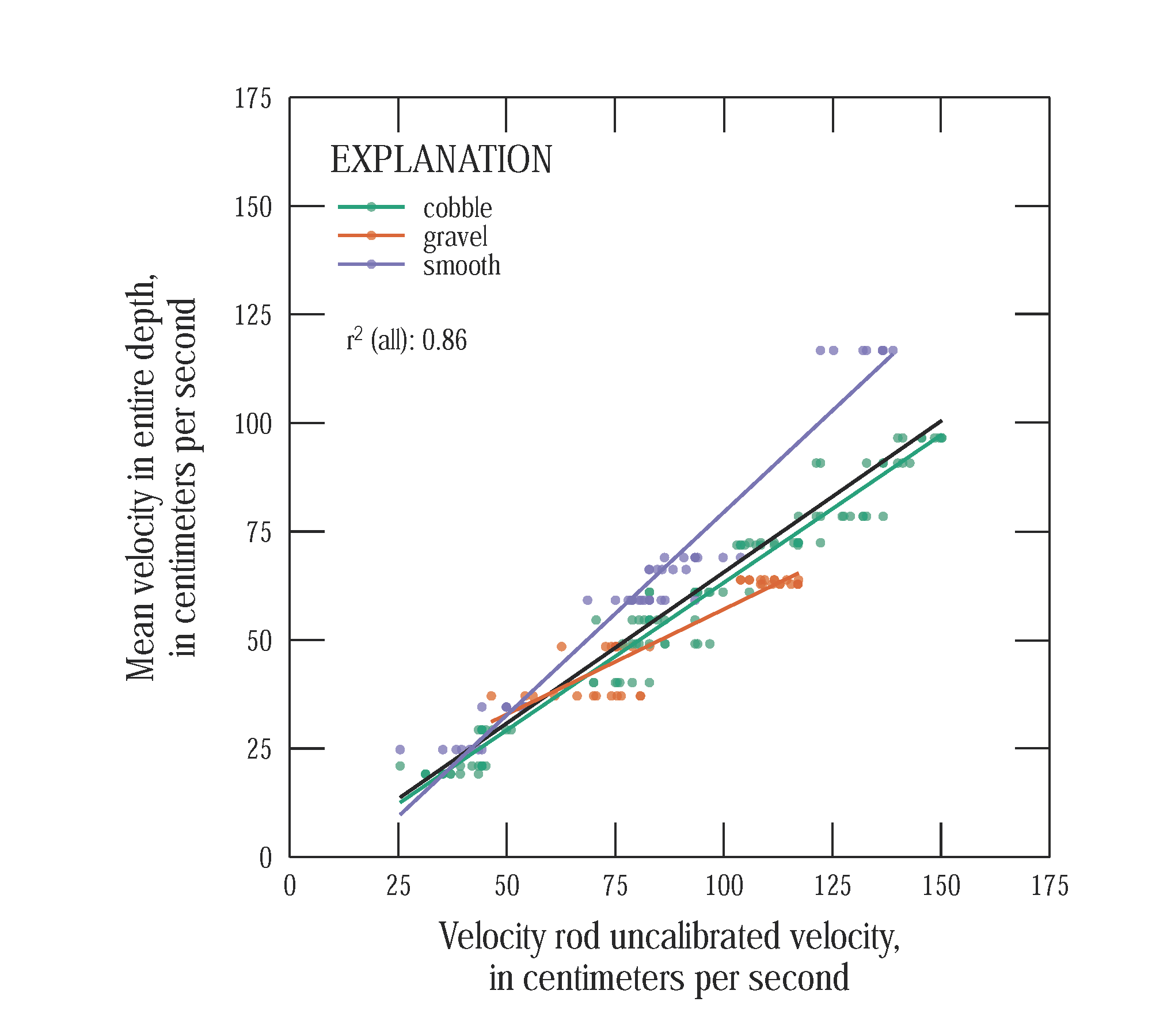

Supplement: S3 Fig — The colored lines are best fit lines for each substrate type and the black line is the best-fit line for all measurements. (TIFF) [file pone.0222263.s004.tiff]

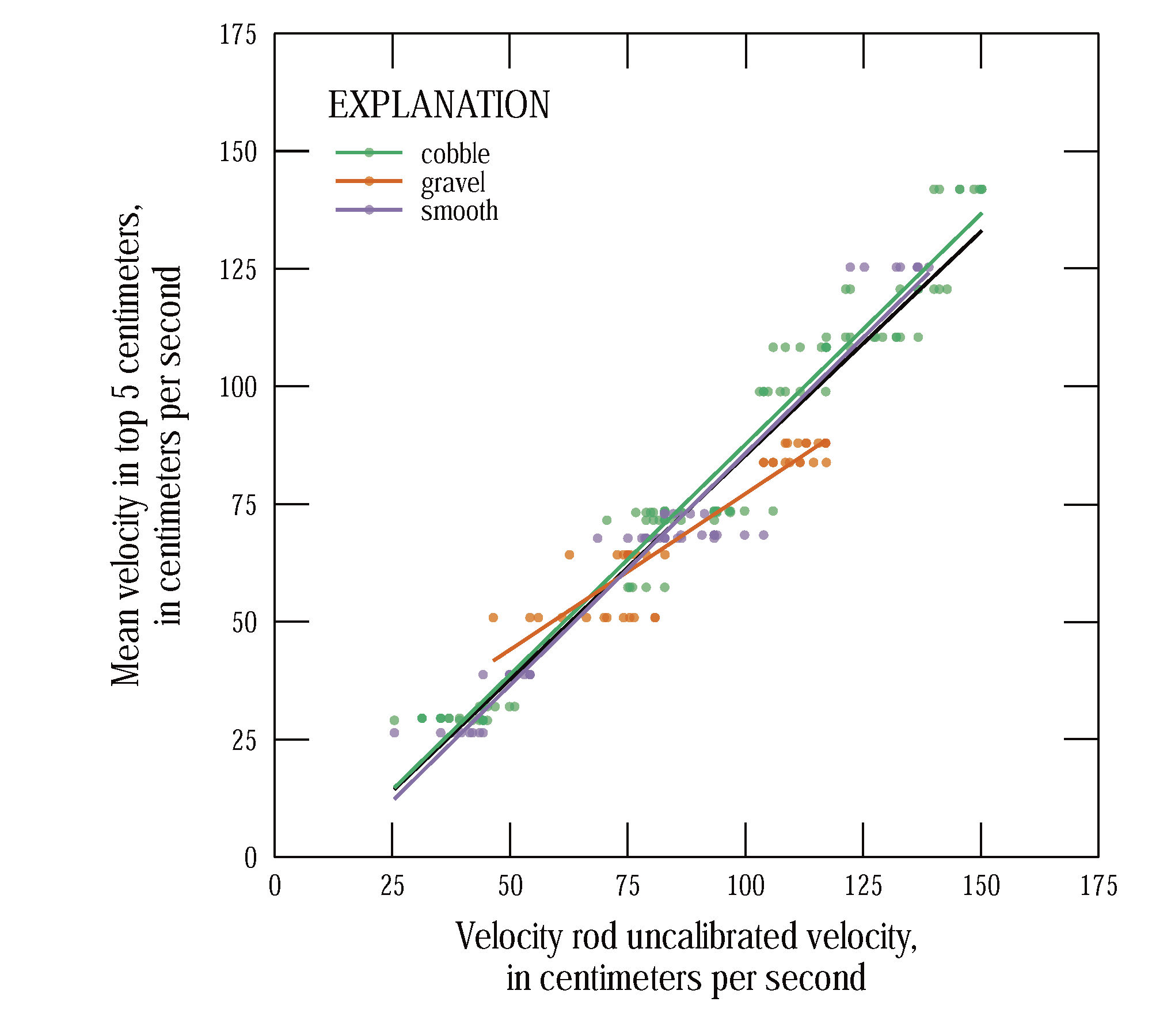

Supplement: S4 Fig — The colored lines are best fit lines for each substrate type and the black line is the best-fit line for all measurements. (TIFF) [file pone.0222263.s005.tiff]

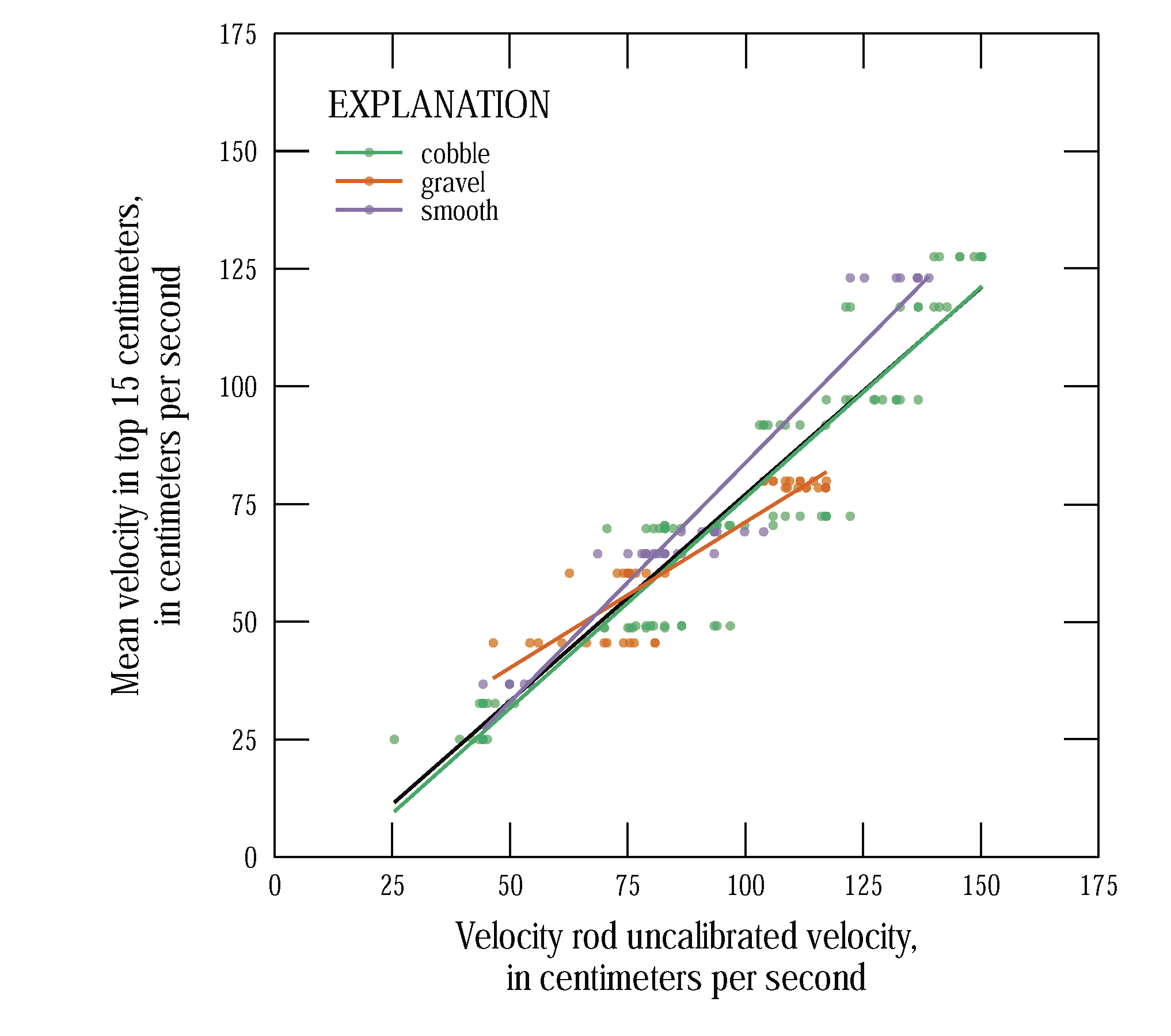

Supplement: S5 Fig — The colored lines are best fit lines for each substrate type and the black line is the best-fit line for all measurements. (TIFF) [file pone.0222263.s006.tiff]
